# Antieosinophil Antibodies Alone or in Combination with Antineutrophil Cytoplasmic Antibodies (ANCA) Detected in Different Autoimmune Conditions

**DOI:** 10.1155/2023/5980287

**Published:** 2023-04-26

**Authors:** Régis Dieckmann, Rille Pullerits, Johan Bylund, Anna Karlsson-Bengtsson, Hans Herlitz, Christine Wennerås, Pontus Thulin

**Affiliations:** ^1^Department of Rheumatology and Inflammation Research, Institute of Medicine at the Sahlgrenska Academy, University of Gothenburg, Gothenburg, Sweden; ^2^Clinical Immunology and Transfusion Medicine, Sahlgrenska University Hospital, Gothenburg, Sweden; ^3^Department of Oral Microbiology and Immunology, Institute of Odontology at Sahlgrenska Academy, University of Gothenburg, Gothenburg, Sweden; ^4^Department of Biology and Biological Engineering, Chalmers University of Technology, Gothenburg, Sweden; ^5^Department of Molecular and Clinical Medicine, Institute of Biomedicine at Sahlgrenska Academy, University of Gothenburg, Gothenburg, Sweden; ^6^Institute of Biomedicine, Department of Infectious Diseases, Sahlgrenska Academy, University of Gothenburg, Gothenburg, Sweden; ^7^Department of Clinical Microbiolog, Sahlgrenska University Hospital, Gothenburg, Sweden

## Abstract

Circulating antieosinophil antibodies (AEOSA) have been associated with various autoimmune conditions affecting the liver, kidneys, lungs, and joints but are not part of routine clinical diagnostics. While analyzing human sera for antineutrophil cytoplasmic antibodies (ANCA) by indirect immunofluorescence (IIF) on granulocytes, 0.8% of analyzed samples were found to be reactive with eosinophils. Our aim was to determine the diagnostic relevance and antigenic specificity of AEOSA. AEOSA were seen either in combination with an myeloperoxidase (MPO)-positive p-ANCA (44%; AEOSA+/ANCA+) or on their own (56%; AEOSA+/ANCA−). AEOSA/ANCA positivity was seen in patients with thyroid disease (44%) or vasculitis (31%), while AEOSA+/ANCA− pattern was more common in patients with autoimmune disorders of the gastrointestinal tract and/or liver. Eosinophil peroxidase (EPX) was the main target recognized in 66% of the AEOSA+ sera by enzyme-linked immunosorbent assay (ELISA). Eosinophil cationic protein (ECP) and eosinophil-derived neurotoxin (EDN) were also identified as target antigens but less frequently and only in combination with EPX. In conclusion, we confirmed that EPX is a major target of AEOSA, illustrating the high antigenic potential of EPX. Our results also demonstrate the presence of concomitant AEOSA/ANCA positivity in a defined patient group. Further research should aim to elucidate the association of AEOSA with autoimmunity.

## 1. Introduction

Indirect immunofluorescence (IIF) tests are routinely used to assess different types of autoantibodies to aid in the diagnosis of autoimmune diseases. Detection of antineutrophil cytoplasmic antibodies (ANCA) by IIF has been part of the diagnostic algorithm of ANCA-associated vasculitis (AAV). To this end, total polymorphonuclear leukocytes are ethanol-fixed on glass slides, incubated with patient sera, and examined for a staining pattern that are most often perinuclear (p-ANCA) or cytoplasmic (c-ANCA) in character. Although the seeded polymorphonuclear leukocytes mainly consist of neutrophils, eosinophils may also be present and can be identified by their bilobed nucleus and red autofluorescence [1]. Antibodies recognizing only eosinophils—antieosinophil antibodies (AEOSA)—were first described by Dolman et al. [2]. The target antigen of AEOSA was shown to be the cytoplasmic protein eosinophil peroxidase (currently abbreviated EPX, formerly designated EPO), and observed in 1% of the patients (8/876) tested for ANCA by IIF. AEOSA were found in conjunction with various autoimmune conditions affecting the kidneys, lungs, and joints [2]. To our knowledge, the study by Dolman et al. is the only one visualizing AEOSA on ANCA slides by IIF. Two other studies have evaluated the presence of anti-EPX antibodies by enzyme-linked immunosorbent assay (ELISA) [3, 4]. Takiguchi et al. [3] detected anti-EPX autoantibodies in autoimmune liver diseases, e.g., primary biliary cholangitis (PBC) (53% of 61 cases), autoimmune hepatitis (AIH) (29% of 31 cases), as well as in chronic viral hepatitis (8% of 87 cases). Mukherjee et al. [4] described anti-EPX reactivity in the sputum of patients with severe eosinophilic asthma. The clinical significance of AEOSA is still unclear. Although anti-EPX autoantibodies have occasionally been found together with antinuclear antibodies (ANA) or antimitochondrial antibodies (AMA) in PBC and AIH, their titers did not correlate with ANA or AMA levels [3]. Furthermore, in the abovementioned studies, anti-EPX autoantibodies were not described together with autoantibodies against the major ANCA target antigens such as myeloperoxidase (MPO), proteinase 3 (PR3), or human neutrophil elastase (HNE) [2, 3]. Finally, purified anti-EPX antibodies have been shown to trigger cell activation, measured either by production of reactive oxygen species (ROS) [2] or eosinophil degranulation [4], implying a pathogenic function for these autoantibodies. Despite their early characterization in 1993, the detection of AEOSA is not a part of clinical routine diagnostics. The aim of this study was to determine the diagnostic relevance and antigenic specificity of AEOSA.

## 2. Materials and Methods

### 2.1. Patient Samples

Blood samples, sent for routine ANCA IIF analysis to the Clinical Immunology Laboratory at the Sahlgrenska University Hospital in Gothenburg, Sweden, were evaluated for the presence of AEOSA by IIF. Patients with positive AEOSA were identified, asked for consent to participate in this study, and followed-up regarding their clinical diseases and symptoms. In total, 36 patients whose sera tested positive for AEOSA by IIF were included in this study. The patients answered questionnaire regarding concomitant diseases, including allergies, current disease symptoms and medication, and heredity for autoimmune diseases, and smoking habits and medical records were carefully reviewed (P.T. and R.P.). The patients were followed-up for 1 year. The patients' characteristics are given in Tables 1–3.

This study protocol was reviewed and approved by the Ethical Review Board of Gothenburg, Sweden. Written informed consent was obtained from all subjects prior to enrollment in this study. This study was carried out in accordance with relevant Swedish guidelines and regulations of Good Clinical Practice were followed.

### 2.2. Laboratory Analyses

Antibodies to thyroperoxidase (TPO) and thyroglobulin (TG) were measured using random access analyzer based on the Sensotronic Memorized Calibration (SMC®) Technology (Alegria, Orgentec Diagnostica GmbH, Mainz, Germany) and serum levels of eosinophil cationic protein (ECP) using Phadia EliA™ Methodology (ImmunoCAP Phadia 250, Thermo Fisher Scientific Inc., Massachusetts, USA) according to manufacturer's instructions.

IgG antibodies against liver-specific antigens were analyzed using a line blot assay (EUROLINE Profile Autoimmune Liver Diseases, Euroimmun AG, Lübeck, Germany) containing the following autoantigens: AMA-M2 (pyruvate dehydrogenase complex), M2-3E (BPO, fusion protein of the E2 subunits of the *α*-2-oxoacid dehydrogenases of the inner mitochondrial membrane), Sp100 (nuclear granula protein), PML (promyelocytic leukemia) protein, gp210 (integral protein of the nuclear membrane), LKM-1 (liver–kidney microsomes, cytochrome P450IID6), LC-1 (liver cytosolic antigen type 1; formiminotransferase cyclodeaminase), SLA/LP (soluble liver antigen/liver–pancreas) antigen, and SSA/Ro52. The assay was carried out using a fully automated EUROBlotOne device (Euroimmun AG, Lübeck, Germany). Blot strips were digitalized using a camera and band intensities were determined by a computer program (EUROLineScan, Euroimmun), with the band intensity thresholds recommended by the manufacturer: signal strengths >15 arbitrary units (AU) were considered positive; 7–14 AU were considered borderline.

### 2.3. ANCA IIF Detection

The presence of ANCA in sera was determined using the IIF ANCA Slide Kit (Immuno Concepts, Sacramento, CA, USA) according to the manufacturer's instructions. If not stated otherwise, sera were diluted 1 : 20 in sample diluent. Serum reactivity to MPO and PR3 was analyzed by using Wieslab® MPO-ANCA and PR3-ANCA capture ELISA kits, respectively, according to the manufacturer's instructions (Euro Diagnostica, Malmö, Sweden). The MPO kit is standardized against the AF-CDC international standard (Reference Human Serum 15, code IS2720).

### 2.4. In-House ELISA for the Detection of AEOSA

Purified human proteins such as EPX (Aviva Systems Biology, San Diego, USA), ECP (Diagnostics Development, Uppsala, Sweden), eosinophil-derived neurotoxin (EDN) (Diagnostics Development), and MPO (Aviva Systems Biology) were used. No cross-reactivity was observed between eosinophil proteins as EPX was not recognized by rabbit (DAKO, A0398) or mouse (DAKO, MPO-7) anti-MPO antibodies, and MPO was not recognized by a mouse anti-EPX antibody (Abcam, AHE-1). ECP and EDN did not contain detectable amount of EPX at the coating concentrations used. EPX and MPO were judged to be natively folded as both proteins retained their peroxidase activity (data not shown). Sera were diluted one-fifth in phosphate-buffered saline (PBS) supplemented with 0.02% NaN_3_, centrifuged at 13,500 *g* in a cooled microfuge, and the supernatants were aliquoted and snap-frozen until further use. All purified proteins were diluted in PBS and used at either 250 ng/mL (EPX and MPO) or at 500 ng/mL (ECP and EDN) for overnight coating of sealed Nunc MaxiSorp 96-well plates (100 *µ*L diluted protein/well, 4°C). Bovine serum albumin (BSA) at a concentration of 500 ng/mL was always run in parallel as a negative control. On the next day, the plates were blocked using 1% BSA in PBS for 1 hr. After one rinse in PBS, prediluted and thawed sera were incubated in duplicate at 1/100 end dilution in 1% BSA in PBS (100 *µ*L/well). Plates were washed three times in 0.1% Tween-20 PBS (PBS-T). Secondary AP-linked goat anti-human IgG (1.3 mg/mL, Sigma, A9544) antibody was added at 1/5,000 dilution in 1% BSA in PBS and incubated for 1 hr (100 *µ*L/well). The substrate para-nitrophenylphosphate (pNPP) (Sigma, S0942) was added (100 *µ*L/well) at a concentration of 1 mg/mL in 1 M diethanolamine, pH 9.8, supplemented with 0.5 mM MgCl_2_. Signal intensity was read at optical density (OD) of 405 nm after 40 min. Net signal intensity was calculated by subtracting the BSA background. The cutoff was calculated as the average signal intensity plus three standard deviations (SD) of control sera obtained from 10 healthy blood donors.

Normalization between assays was performed by setting one reference serum to 100% (EPX ELISA: patient E3; ECP ELISA: patient E9; MPO ELISA: patient AMPO6). The ratio between two reference sera had to be in a constant range for the assay to be accepted (EPX ELISA: E3/E4 = 40%; ECP ELISA: E9/E10 = 40%; MPO ELISA: AMPO6/AMPO7 = 33%). SDs for assay inter- and intravariability were <12%. For the EPX ELISA, variability between two duplicates was 7.2% ± 4.6%.

### 2.5. Confocal Microscopy Analysis

Björnsdottir et al. [5] described that granulocytes from healthy blood donors were purified from buffy coats. Cells were suspended in ice-cold PBS at 1.5 million cells/mL and allowed to attach to 12-well adhesion slides (Marienfeld, Germany) at 4°C for 5 min. Cells were fixed in 3.5% paraformaldehyde in PBS for 10 min at 4°C, followed by 5 min at room temperature. Samples were blocked in 1% BSA in PBS at room temperature, rinsed in PBS, and incubated with prediluted patient sera (see ELISA method section) at an end dilution of 1/50 for 1 hr. Samples were then washed three times for 5 min in PBS, incubated with goat anti-human IgG Alexa 488 (Life Technologies, H10120) at 1/300 dilution in 1% BSA-PBS for 1 hr. After two 5 min PBS washes, samples were stained with 4′,6-diamidino-2-phenylindole dihydrochloride (DAPI), diluted 1/10,000 in PBS from a 5 mg/mL stock solution for 10 min, washed for 5 min in PBS, and mounted in fluorescence mounting medium (DAKO, S3023). Cells were visualized with 63x oil objective using a confocal Zeiss LSM700 microscope.

### 2.6. Statistical Analysis

Statistical analyses were performed using GraphPad Prism version 9.2.0 (GraphPad Software, La Jolla, California, USA). Differences between groups were assessed by the Mann–Whitney *U* test. Differences in frequency were calculated using the *χ*^2^ or Fisher's exact test as appropriate. *p*-value < 0.05 was considered as statistically significant.

## 3. Results

### 3.1. Detection of AEOSA by Immunofluorescence

**Figure 1:**
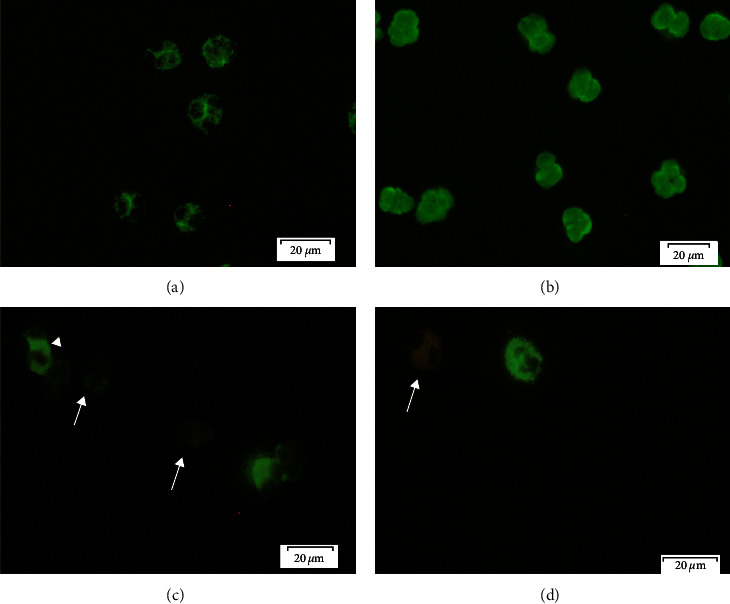
Representative immunofluorescence (IF) images of common antineutrophil cytoplasmatic antibody (ANCA) patterns: (a) a cytoplasmic staining pattern (c-ANCA) obtained with serum from antiproteinase-3-positive vasculitis patient; (b) a perinuclear staining pattern (p-ANCA) obtained with serum from antimyeloperoxidase-positive vasculitis patient; (c) an uncommon IF pattern seen in ANCA IF slides representing antieosinophil antibodies (indicated with arrowheads) and negative neutrophils (indicated with arrows); (d) the cells with the two-lobed nuclear appearance and the red autofluorescence of the cell cytoplasm by IIF microscopy represent eosinophils (indicated with an arrow). The stained cell nearby is a neutrophil.

**Figure 2:**
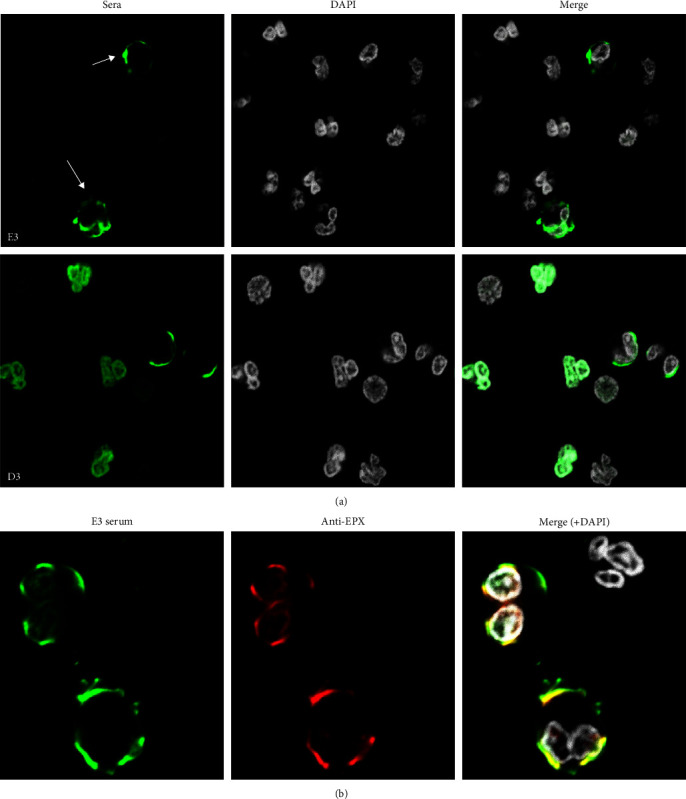
(a) Typical immunofluorescence images of autoimmune reactivity against eosinophils for AEOSA+/ANCA− and AEOSA+/ANCA+ sera. The p-ANCA staining visible on neutrophils in AEOSA+/ANCA+ sera (D3) is absent in AEOSA+/ANCA− sera (E3) where only the eosinophils (arrow) are stained. Scale bar = 20 *µ*m; (b) a representative picture of colocalization of the signals of anti-EPX-positive serum from patient E3 (green) and mouse anti-EPX monoclonal (red) in confocal microscopy.

The Clinical Immunology Laboratory at Sahlgrenska University Hospital serves the Region Västra Götaland of 1.6 million inhabitants. Approximately 2,500–3,000 ANCA IIF analyses are performed yearly using ANCA slides prepared from ethanol-fixed whole granulocyte pellets containing an average eosinophil/neutrophil ratio of 1/30. Out of these 2,500–3,000 sera, 70%–75% are negative for ANCA, 8%–10% stain for p-ANCA (Figure 1(b)), and 4%–6% stain positive for c-ANCA (Figure 1(a)). In the remaining cases, atypical ANCA or suspected ANA are found. In some patients, we observed that other cells than neutrophils were stained on the ANCA slides either alone (Figure 1(c)) or together with p-ANCA (Figure 2(a)). These cells could be distinguished from ANCA staining by the two-lobed nuclear appearance and the red autofluorescence of the cell cytoplasm by IIF microscopy and were suspected to be eosinophils (Figure 1(d)). This was further confirmed by confocal microscopy using antibodies to EPX together with patient sera (Figure 2(b)) Altogether, 0.8% of serum samples subjected to ANCA IIF-stained eosinophils. These AEOSA were seen in combination with p-ANCA (16/36, 44%; AEOSA+/ANCA+) or in absence of ANCA (20/36, 56%; AEOSA+/ANCA−) but never together with c-ANCA. The target antigens of AEOSA were always cytoplasmic as observed by confocal immunofluorescence (Figure 2) and were hypothesized to be localized in eosinophilic granules.

### 3.2. Eosinophil Peroxidase (EPX) Is the Main Antigen Recognized by AEOSA

**Figure 3:**
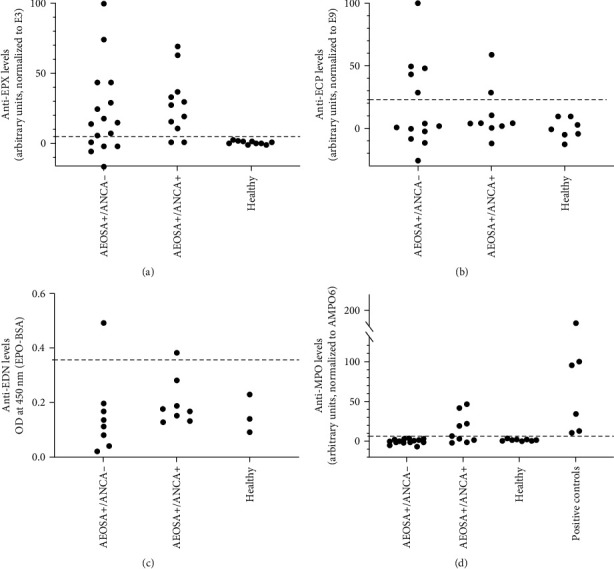
Titers of antibodies against (a) eosinophil peroxidase (anti-EPX), (b) eosinophil cationic protein (anti-ECP), (c) eosinophil-derived neurotoxin (anti-EDN), and (d) myeloperoxidase (anti-MPO) as determined by ELISA at study inclusion. The antibody titers are expressed as arbitrary units at OD 450 nm normalized to a reference serum. Sera from patients that tested positive for AEOSA in the IIF staining were analyzed; E-group refers to sera from AEOSA+/ANCA− patient samples and D-group refers to AEOSA+/ANCA+ patient samples. The cutoff limit (indicated with dashed line) was set at +3 SD above the average signal in serum samples from healthy controls.

Next, we sought to determine the antigenic specificity of AEOSA by developing an in-house ELISA method specific for three major cytoplasmic granule proteins of eosinophils—EPX, ECP, and EDN. Serum samples from 27 patients were available for this analysis, either as a sample at inclusion (*n* = 22) together with a follow-up sample (*n* = 16) or only as a sample at 1-year follow-up (*n* = 5). The main autoantigen recognized by AEOSA was EPX (18/27, 66%) (Figure 3(a)). Anti-EPX antibodies were detected in 9 out of 11 (82%) of the AEOSA+/ANCA+ sera and in 11 out of 16 (69%) of the AEOSA+/ANCA− sera. The AEOSA IIF staining pattern on neutrophils colocalized with EPX staining (Figure 2).

The eosinophil antigens ECP and EDN were also identified as target antigens of AEOSA (Figures 3(b) and 3(c)) but were less frequently detected; anti-ECP was seen in 30% (*n* = 9) and anti-EDN was seen in 14% (*n* = 2) of the AEOSA+ sera and were present only in combination with anti-EPX antibodies (Tables 1 and 2). Anti-EDN antibodies were detected only in anti-EPX/ECP double-positive sera. Almost one-third of the AEOSA+/ANCA− sera (5/16) and every fifth AEOSA+/ANCA+ serum (2/11) did not show any reactivity in ELISA against EPX, ECP, or EDN but still reacted with eosinophils in IIF, indicating that they recognize other eosinophil antigens.

Only AEOSA+/ANCA+ sera were positive in the anti-MPO ELISA, whereas sera from healthy controls and AEOSA+/ANCA− patients displayed no anti-MPO reactivity (Figure 3(d)).

### 3.3. Variation of Antibody Titers over Time

**Figure 4:**
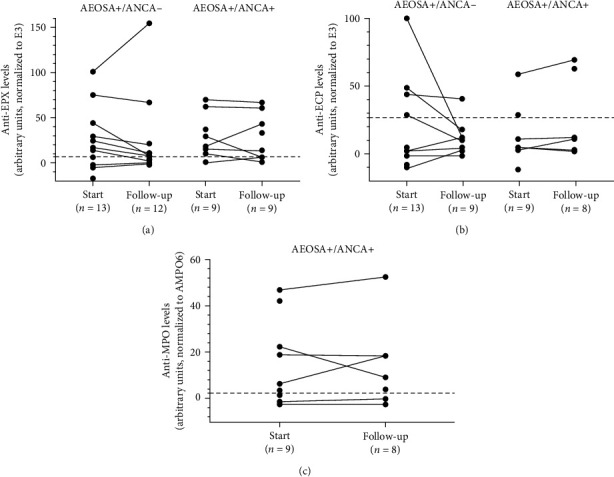
Antibody titers at study inclusion and at the first follow-up as determined by ELISA against (a) eosinophil peroxidase (anti-EPX), (b) eosinophil cationic protein (anti-ECP), and (c) myeloperoxidase (anti-MPO). The antibody titers are expressed as arbitrary units at OD 450 nm normalized to a reference serum. E-group refers to sera from AEOSA+/ANCA− patient samples and D-group refers to AEOSA+/ANCA+ patient samples. The cutoff limit (indicated with dashed line) was set at +3 SD above the average signal in healthy controls.

At 1-year follow-up, autoantibodies against EPX were still detectable by ELISA in 14/21 (67%) of patients. The anti-EPX antibody titers had decreased in 6/10 (60%) of the AEOSA+/ANCA− group and in 2/7 (29%) of the AEOSA+/ANCA+ group of the patients with available follow-up sera (Figure 4(a)). Anti-EPX titers stayed stable in five patients and increased in two patients.

Although anti-ECP reactivity was detected only in combination with anti-EPX reactivity, the variation in the antibody titers over time did not correlate, neither was there a correlation between anti-EPX and anti-MPO titers in the double-positive sera over time (Figures 4(b) and 4(c)).

Besides EPX and MPO, TPO is another peroxidase with a high antigenic potential. However, anti-TPO reactivity was not correlated to any of the tested parameters (Tables 2 and 3).

### 3.4. Clinical Symptoms and Diseases in Patients with AEOSA

There was no significant age difference between the AEOSA+/ANCA− single-positive group and the AEOSA+/ANCA+ double-positive group (mean age 59 ± 18 vs. 55 ± 17 years, respectively). Women were overrepresented in both groups: 65% and 75%, respectively.

The clinical symptoms associated with the presence of AEOSA in our cohort varied. The majority (75%) of patients with AEOSA had one or several inflammatory or autoimmune diseases affecting the liver, kidneys, thyroid gland, gastrointestinal tract, and/or joints (Tables 2 and 3).

AEOSA+/ANCA+ patients significantly more often had thyroid diseases (7/16, 44%) than AEOSA+/ANCA− patients (2/20, 10%; *p* < 0.05) (Table 1). In addition, vasculitis manifestations were significantly seen more frequent in AEOSA+/ANCA+ patients (6/16, 38%) than in AEOSA+/ANCA− patients (1/20, 5%; *p* < 0.05).

In contrast, AEOSA+/ANCA− patients had a history of pathologies affecting the gastrointestinal tract (5/20, 25%) and/or the liver (6/20, 30%) and/or had positivity or borderline positivity for autoantibodies against liver antigens associated with autoimmune liver diseases (9/20, 45%) (Table 3). One or more of these factors were present in 16/20 (80%) of the AEOSA+/ANCA− patients.

All anti-EPX/anti-ECP double-positive AEOSA+/ANCA− sera (6/6) were found in patients with a history of gastrointestinal tract disease and/or autoimmune disease (with 3/3 cases of ankylosing spondylitis). All anti-EPX/anti-MPO double-positive AEOSA+/ANCA+ sera (5/5) were found in patients with a history of thyroid disease or vasculitis (Tables 2 and 3).

Of note, four out of five patients with available data for blood cell differential counts in AEOSA+ group and one out of five patients in ANCA+/AEOSA+ group had eosinophil counts below the reference value.

## 4. Discussion

In this study, we have identified autoantibodies against eosinophils, AEOSA, and show that they can be found either alone (AEOSA+/ANCA−) or in association with p-ANCA (AEOSA+/ANCA+). As previously unrecognized, these two groups are associated with distinct disease spectra. Besides confirming that EPX is the main antigenic target of AEOSA, we also show that both ECP and EDN are autoimmune targets in eosinophils.

AEOSA, although never in combination with ANCA, have been described in the serum of patients suffering from various autoimmune conditions affecting the liver, kidneys, and joints [2, 3]. However, we could distinguish two groups: the previously described AEOSA+/ANCA− patients and the previously unrecognized AEOSA+/ANCA+ patients.

The majority of AEOSA+/ANCA− serum samples (75%) in this study came from patients with a history of gastrointestinal tract and/or liver inflammation, often on an autoimmune background. These patients correspond to those described in the study by Dolman et al. [2]. In their study, the presence of AEOSA was not associated with any disease and only two patients out of eight (25%) were diagnosed with colitis with or without chronic hepatitis. However, an association of anti-EPX antibodies with autoimmune liver disease has been shown by Takiguchi et al. [3], but a connection to gastrointestinal inflammation was not studied. In this regard, the detection of anti-ECP in our cohort may define a more precise patient group as all AEOSA+/ANCA− sera displaying both anti-EPX and anti-ECP reactivity appeared mainly in patients with either gastrointestinal tract inflammation and/or ankylosing spondylitis. None of these patients were positive for antibodies against TPO or liver antigens. Therefore, anti-ECP autoantibodies might be an indicator of gastrointestinal tract inflammation, which in some cases is coupled to ankylosing spondylitis. An inflammatory bowel disease is a recognized extra-articular manifestation in ∼7% of patients with ankylosing spondylitis [6]. Apparently, the physiological triggers driving the production of anti-EPX and anti-ECP are not the same since the anti-ECP and anti-EPX antibody titers sometimes varied independently of each other. Furthermore, anti-ECP antibodies might not play the same role in the disease. Eosinophils are recruited to the colon in clinically active ulcerative colitis and extracellular deposits of eosinophil granule proteins are demonstrated in biopsies of these patients [7]. In rats, treatment with anti-ECP antibodies favors remission in dextran-induced colitis, a general model of colon inflammation with proven accumulation of eosinophil granule proteins [8]. Although increased serum concentrations of ECP have been detected in patients with active ankylosing spondylitis [9], there was no correlation of increased ECP concentration and detection of anti-ECP autoantibodies or anti-ECP antibody titers in this study.

This study is the first study to demonstrate the presence of concomitant AEOSA/ANCA positivity in a defined patient group. AEOSA+/ANCA+ sera were predominantly associated with a patient history of thyroid disease and/or vasculitis. This link was stronger for anti-MPO/anti-EPX double-positive patients as all such patients had a history of thyroid disease or vasculitis. Of note, anti-MPO antibodies are more frequently detected in patients with both AAV and thyroid disease compared to patients affected by AAV only [10, 11]. In the study by Dolman et al. [2], 25% of the EPX-positive patients suffered from thyroid disease [2], whereas the corresponding prevalence in this study was 10%. Despite the presence of autoantibodies against EPX, neither vasculitis nor thyroid disease was reported in the previously studied cohorts [3, 4]. Interestingly, no patients affected by eosinophilic granulomatosis with polyangiitis (EGPA) were found in this study although EGPA is characterized by the formation of granuloma and the recruitment of eosinophils to these granulomas [12].

We also observed that anti-EPX titers fluctuated over time in some patients. Of note, six patients out of eight with decreased levels of anti-EPX at follow-up received anti-inflammatory treatment. Four patients in AEOSA+/ANCA− group and two patients in AEOSA+/ANCA+ group received treatment with prednisolone at diagnosis, whereas three of them in AEOSA+/ANCA− group had also concomitant disease-modifying drug. Furthermore, two patients who displayed increased anti-EPX titers at follow-up had withdrawn the prednisolone treatment. These results suggest that AEOSA might reflect the inflammatory activity of the disease.

This study has some limitations, which need to be taken into account. First, the occurrence of AEOSA is quite rare, about 1% of the selected patient population whose samples were referred to ANCA IIF screening. This scarcity makes it difficult to obtain sufficient study population in order to draw firm conclusions about the clinical relevance of AEOSA. Second, in patients with AEOSA, a diversity of different heterogeneous diseases is recognized. Third, this study had an observational prospective nature and we could not interfere with the treatment and diagnostic procedures. As a result, some of the laboratory analyses, e.g., cell differential counts, including eosinophils, were not available in all patients.

In conclusion, the presence of AEOSA observed by IIF is the manifestation of autoimmune reaction against EPX, ECP, EDN, or against other eosinophilic antigens such as galectin-10 or major basic protein, which we did not evaluate in this study. Our observations suggest that AEOSA are often accompanied with other autoantibodies and can be found in patients with liver or gastrointestinal disease when found alone or in patients with vasculitis or thyroid disease when found together with p-ANCA. While anti-EPX is the main antigenic target of AEOSA, anti-ECP seems to be found in rare cases of ulcerative colitis and ankylosing spondylitis. However, the results should be interpreted cautiously and further studies are needed to evaluate the clinical relevance of AEOSA.

## Figures and Tables

**Table 1:** Clinical and laboratory characteristics of AEOSA+/ANCA+ and AEOSA+/ANCA− patients.

	AEOSA+/ANCA+ (*n* = 16)	AEOSA+/ANCA− (*n* = 20)	*p*-values
Age, years ± SD	59 ± 18	55 ± 17	0.3725
Female, *n* (%)	12 of 16 (75)	13 of 20 (65)	0.8125
TPO positive, *n* (%)	6 of 15 (40)	2 of 18 (11)	0.1015
TG positive, *n* (%)	2 of 5 (40)	1 of 7 (14)	0.5227
SMA positive, *n* (%)	2 of 12 (17)	2 of 18 (11)	0.9999
Parietal cell antibody positive, *n* (%)	4 of 12 (33)	0 of 18 (0)	**0.0181**
Diagnosis of liver disease, *n* (%)	1 of 16 (6)	5 of 20 (25)	0.1962
Diagnosis of thyroid disease, *n* (%)	7 of 16 (44)	2 of 20 (10)	**0.0491**
Diagnosis of vasculitides, *n* (%)	6 of 16 (38)	1 of 20 (5)	**0.0298**

Abbreviations: TPO, thyroid peroxidase; TG, thyroglobulin; SMA, smooth muscle antibody. Significant *p*-values have been highlighted in bold.

**Table 2:** The clinical characteristics and disease profile of the AEOSA+/ANCA+ individual patients.

	Age (years)	Sex	Anti-TPO	Anti-TG	Anti-MPO	SMA/AMA/Par (IF)	Liver antibodies	AEOSA specificity	Disease history		
									GI/Liver disease	Thyroid disease	Other diseases
D1	66	M	Negative		Positive	Negative	Negative	EPX+			Vasculitis (MPA)
D2	57	M	Negative		Positive	Negative	Negative	EPX+			Kidney disease unspecified
D3	48	F	Negative	Negative	Positive	n.a.	Negative	EPX borderline	Liver steatosis	Hyperthyroidism	Fibromyalgia, vitiligo, pulmonary embolism
D4	18	F	Negative		Positive	Negative	Negative	EPX−			Vasculitis (GPA)
D5	69	F	Negative	Negative	Positive		Negative	EPX+			Vasculitis (MPA), breast cancer, pituitary tumor, history of alcohol abuse
D6	71	M	n.a.		Positive	n.a.	n.a.	n.a.			
D7	77	F	Negative		Positive	Negative	Negative	EPX+ ECP+ EDN+			Chronic laryngitis, chronic rhinitis, sinusitis, duodenitis, COPD, diabetes type II
D8	70	F	Negative		Positive	Par 640	Negative	n.a.	Atrophic gastritis	Thyrotoxicosis	Nonallergic asthma, primary hyperparathyroidism, polyneuropathy
D9	70	F	Negative		Negative	SMA 80	Negative	EPX+ ECP+	Ulcerative colitis	Hypothyroidism	Lichen planopilaris, diabetes type II, chronic kidney failure
D10	80	M	Borderline	Borderline	Positive	Par 80	LC-1(+) gp210+ 3E(BPO)+	EPX+		Hypothyroidism	MDS, vasculitis (giant cell arthritis)
D11	43	F	Positive		Positive	Negative	SLA/LP+ gp210+ PML+ LC-1(+)	EPX+		Hyperthyroidism	Ehler–Danlos syndrome, asthma
D12	36		Positive		Positive	n.a.	n.a.	EPX+		Hashimoto thyroiditis	Vasculitis (IgA nephritis)
D13	66	F	Positive	Positive	Positive	Par 80	M2+	EPX+ ECP+	Pernicious anemia	Hashimoto thyroiditis	Diabetes mellitus type I, vitiligo, skin vasculitis/urticaria
D14	36	F	Borderline		Positive	Negative	Negative	n.a.			SLE, pericarditis, urticaria
D15	71	F	Positive		Positive	SMA 80	LKM-1(+)	n.a.			Recurrent urticaria, allergic rhinitis/asthma
D16	71	F	Negative	Negative	Negative	Par 640	LC-1(+) LKM-1(+)	n.a.			Pericarditis, palmoplantar psoriasis

Liver antibody profile tested includes AMA, AMA-M2, 3E (BPO), PML, Sp100, gp210, LKM-1, LC-1, and SLA/LP. Abbreviations: TPO, thyroid peroxidase; TG, thyroglobulin, MPO, myeloperoxidase; IF, immunofluorescence; SMA, smooth muscle antibody; AMA, antimitochondrial antibodies; Par, parietal cell antibodies; AEOSA, antieosinophil cytoplasmic antibodies; GI, gastrointestinal; EPX, eosinophil peroxidase; ECP, eosinophil cationic protein; EDN, eosinophil-derived neurotoxin; MPA, microscopic polyangitis; GPA, granulomatosis with polyangiitis; COPD, chronic obstructive pulmonary disease; SLE, systemic lupus erythematosus; n.a., not available.

**Table 3:** The clinical characteristics and disease profile of the AEOSA+/ANCA− individual patients.

	Age (years)	Sex	Anti-TPO	Anti-TG	SMA/AMA/Parietal IF	Liver antibodies	AEOSA	Disease history		
								GI/Liver disease	Thyroid disease	Other diseases
E1	69	F	Borderline		Negative	Negative	EPX+ ECP+	Ulcerative colitis	Hypothyroidism	AS, pustular psoriasis
E2	75	M	Negative		SMA 80	Negative	EPX+	Pernicious anemia	Hyperthyroidism	AIHA, alcohol abuse
E3	54	F	Positive	Positive	SMA 80	Negative	EPX+			Asthma, obesity
E4	73	F	Negative		Negative	SLA/LP(+) PML(+) 3E(BPO)(+)	EPX+ ECP+ EDN+	Collagenous colitis		
E5	54	F	Negative	Negative	n.a.	3E(BPO)(+)	EPX−	Liver fibrosis		HCV
E6	81	M	n.a.		Negative	SLA/LP(+) PML(+) 3E(BPO)(+)	EPX−			Obesity, RA, calcitonin hypersecretion
E7	36	M	Negative		Negative	Negative	EPX−	Liver fibrosis		Kidney Tx (hereditary nephropathy)
E8	64	F	Negative		Negative	3E(BPO)(+)	EPX−			B-cell lymphoma, bilateral uveitis
E9	78	M	Negative	Negative	Negative	Negative	EPX+ ECP+	Suspected liver fibrosis		MDS
E10	35	F	Negative		Negative	PML(+) 3E(BPO)(+)	EPX+ ECP+			AS, MCTD
E11	53	M	Negative		Negative	PML(+) LC-1(+)	EPX+			Alcohol abuse, CHD, obesity, kidney failure
E12	53	F	Negative		Negative	PML(+) 3E(BPO)(+)	EPX+ ECP+	Ulcerative colitis,liver failure episode		AS, fibromyalgia, allergic asthma/rhinitis
E13	54	F	Negative		Negative	Negative	EPX+	Liver Tx		Chronic kidney failure, APS-1
E14	47	F	Negative		Negative	Negative	EPX+			Chronic sinusitis, asthma
E15	62	F	Negative	Negative	Negative	Negative	EPX+			Kidney sclerosis, alcohol abuse, primary parathyroidism
E16	49	M	Negative	Negative	Negative	Negative	EPX−	Ulcerative colitis, PBC—liver Tx		
E17	30	M	Negative	Negative	Negative	n.a.	n.a.			Vasculitis (IgA nephritis)
E18	42	F	Negative		Negative	M2(+) gp210++ LKM-1+ LC-1(+) SLA+	n.a.			Fibromyalgia, asthma
E19	23	F	n.a.		n.a.	n.a.	n.a.			
E20	70	F	Negative	Negative	Negative	LC-1(+)	n.a.			Emphysema

Liver antibody profile tested includes AMA, AMA-M2, 3E (BPO), PML, Sp100, gp210, LKM-1, LC-1, and SLA/LP. For anti-TG only, known results are indicated. Abbreviations: M, male; F, female; TPO, thyroid peroxidase; TG, thyroglobulin; MPO, myeloperoxidase; IF, immunofluorescence; SMA, smooth muscle antibody; AMA, antimitochondrial antibodies; Par, parietal cell antibodies; AEOSA, antieosinophil cytoplasmic antibodies; GI, gastrointestinall Tx, transplanted; AS, ankylosing spondylitis; AIHA, autoimmune hemolytic anemia; HCV, hepatitis C virus; RA, rheumatoid arthritis; MDS, myelodysplastic syndrome; MCTD, mixed connective tissue disease; CHD, coronary heart disease; APS-1, autoimmune polyendochrine syndrome-1; n.a., not available.

## Data Availability

The original data presented in this study are included in the article. Further requests can be directed to the corresponding author.
